# 2D-Cross Correlation Spectroscopy Coupled with Molecular Fluorescence Spectroscopy for Analysis of Molecular Structure Modification of Camel Milk and Cow Milk Mixtures during Coagulation

**DOI:** 10.3390/foods9060724

**Published:** 2020-06-02

**Authors:** Oumayma Boukria, El Mestafa El Hadrami, Shaxnoza Sultanova, Jasur Safarov, Françoise Leriche, Abderrahmane Aït-Kaddour

**Affiliations:** 1 Applied Organic Chemistry Laboratory, Sciences and Techniques Faculty, Sidi Mohamed Ben Abedallah University, Route d’Immouzer, Fès BP 2202, Morocco; boukriaoumayma@hotmail.com (O.B.); elmestafa.elhadrami@usmba.ac.ma (E.M.E.H.); 2 Department of Food Engineering, Faculty of Mechanical Building, Tashkent State Technical University Named after Islam Karimov, University str. 2, Tashkent 100095, Uzbekistan; sh.sultanova@yahoo.com (S.S.); jasursafarov@yahoo.com (J.S.); 3 Université Clermont-Auvergne, INRAE, VetAgro Sup, 63370 Lempdes, France; francoise.leriche@vetagro-sup.fr

**Keywords:** milk, mixture, spectroscopy, 2D COS

## Abstract

Synchronous fluorescence spectroscopy (SFS) coupled with two-dimensional correlation spectroscopy (2DCOS) was employed to monitor, at the molecular level, the coagulation of five mixture ratios of camel’s milk (CaM) and cow’s milk (CM) (100% CaM, 75% CaM:25% CM, 50% CaM:50% CM, 25% CaM:75% CM and 100% CM). The dissimilarities among the different formulations are highlighted on the synchronous 2DCOS-SFS. In addition, according to the cross-peak symbols in synchronous and asynchronous spectra, the rate of response modification in riboflavin, protein and vitamin A matched with common coagulation phenomena usually reported during chymosin coagulation (hydrolysis of κ-casein, destabilization of casein micelles and aggregation). This study demonstrated that 2DCOS-SFS is a successful strategy to discriminate milk mixtures and to monitor molecular structure modifications during coagulation process.

## 1. Introduction

Nowadays, the production of cow’s milk (CM) is dominant, but the original quality of non-bovine milk, such as camel’s milk (CaM), is now better described and is experiencing growing interest [1]. CaM, goat’s milk, ewe’s milk and buffalo’s milk have received special attention because of their recognition as potential functional foods from a nutritional point of view. It is admitted that CaM presents a high nutritional quality; e.g., it has three times more vitamin C, minerals (e.g., K^+^, Cu^2+^ and Mn^2+^), and essential and polyunsaturated Fatty Acids (FAs) than CM [2,3]. It is also considered as exhibiting properties to manage chronic ailments [4], e.g., tuberculosis, jaundice and asthma. Thus, mixing milks from different species appears as a good strategy to increase the consumption of non-bovine milk and enable consumers and dairy companies to benefit from their nutritional and technological advantages. This opportunity requires the availability of suitable methods to assess the quality features of products but also to control the different stages of their manufacturing process. Milk coagulation is an important step in the development of texture of different dairy products (e.g., cheeses, yogurts). Therefore, a good management of this operation is essential to obtain a cheese with a good texture. Among other factors, it was reported that texture features of cheese are affected by their structural properties, composition and fat distribution that are highly dependent on the making process [5]. The management of the coagulation is also important because it was reported that the microstructure of dairy products is closely related to other quality features of cheeses, like physicochemical properties, flavor, color, nutritional profile and bioavailability of nutrients [6]. 

Dairy products contain natural fluorophores like Maillard reaction products, amino acid groups (e.g., tryptophan, tyrosine), vitamins (riboflavin and vitamin A) and nicotinamide adenine dinucleotide (NADH), among others. Each fluorophore presents a specific excitation and emission spectrum that can be used to study structural changes at the molecular level and predict and identify the composition of food products. The presence of these fluorescence molecules is used as an advantage in fluorescence spectroscopy in order to perform non-destructive analysis of dairy products especially when coupled with chemometrics [7].

Different excitation or emission configurations can be used to study food and dairy products. Among these configurations, synchronous fluorescence spectroscopy (SFS) seems very interesting because it is a good compromise between classical fluorescence spectroscopy (fixing the excitation wavelength and recording emission spectrum and vice versa) and total excitation emission matrix (EEM). In the SFS configuration, spectra are recorded after scanning simultaneously the excitation and emission monochromators with a wavelength difference (Δλ) providing a good fingerprint identification of the product fluorophores [8]. In dairy product analysis, SFS was used to discriminate between milk samples [9], to determine the effects of mild heating, acidification and added minerals on structural changes of milk components [7,10,11] and to study effects of salts and heating on cheese molecular structure [12,13,14].

The understanding of the impact of the association of milk from different species on molecular structure, location and interactions of components (e.g., fat, proteins) during manufacture is essential for predicting quality attributes of dairy products and particularly cheese. However, it is generally difficult to describe the milk coagulation by using fluorescence spectroscopy from conventional one-dimension fluorescence spectra (i.e., wavelength vs. fluorescence intensity) or by using conventional chemometric analytical methods (e.g., principal component analysis and partial least squares discriminant analysis). 

The concept of 2D correlation spectroscopy (2D COS) was first introduced by Noda [15,16] and later extended to the generalized 2D correlation spectroscopy [17]. The generalized 2D COS is a useful technique that has been developed to deal with signal fluctuations induced by different external perturbation factors, like time, or any other physical variables such as temperature or pressure [17,18,19]. Generalized 2D COS is particularly powerful to simplify spectral data analysis due to their general complexity generally assigned to the number of observed bands and their potential overlapping. This method relies on calculating synchronous and asynchronous changes in signal intensities at different wavelengths. This method can be used to unfold spectra on a second dimension in order to increase spectral resolution and to carefully analyze chemical and structural information concerning molecular events [20,21], spectral response sequences, relative intensity changes, and so on [22]. 

Recently, 2DCOS was widely investigated in various areas to deeply analyze fluorescence spectra of, e.g., tissue culture, recycled water systems, and humic substances [23,24,25,26]. Nonetheless, no study evaluated the potential of this method coupled with fluorescence spectroscopy to study the molecular structure modifications occurring during milk coagulation and to discriminate mixture of milk samples from different species during coagulation. Therefore, the present paper will focus on describing coagulation of milk mixtures from CaM and CM by a method combing synchronous fluorescence spectra and 2DCOS. This was performed in order to appraise the development of an accurate, rapid and feasible analytical method to monitor milk coagulation and to distinguish between different formulations of milk during coagulation because this can be useful for identification of milk adulteration.

## 2. Materials and Methods 

### 2.1. Materials 

Raw CM was purchased from a local dairy farm located in Marmilaht, France, while raw CaM was purchased from a local farm located at Fez, Morocco. Milk samples were frozen at −20 °C and forwarded to the laboratory in refrigerated condition. The mean biochemical composition was analyzed with the Gerber method for fat, Kjeldahl method for protein and colorimetric method for lactose according to the normative references (ISO 488:2008, ISO 8968-1:2014, and ISO 26462:2010). The average composition of CM was 3.10% fat, 3.21% protein and 4.16% lactose and 4.77% fat, 2.96% protein and 4.82% lactose for CaM. The samples were maintained at this temperature until analysis. Before the analysis, samples were defrosted at 4 °C for 24 h. Then, five different formulations were prepared by mixing both types of milk in different proportions. Before mixing CaM and CM, samples were warmed to 40 °C (±1 °C) in a water bath and gently mixed for 1 min by hand. The volume fractions (%) of CaM in the different formulations were 100% (i.e., CaM), 75% (i.e., CaM3:CM1, *v*/*v*), 50% (i.e., CaM1:CM1, *v*/*v*), 25% (i.e., CaM1:CM3, *v*/*v*) and 0% (i.e., CM). Based on the recommendation of the manufacturer, milk coagulations were realized at 40 °C (±1 °C) after adding 2.5 μL/mL of commercial rennet (CHY-MAX^®^ M—Chr. Hansen) containing 50 mg/L of chymosin. After adding chymosin, the milk was gently mixed by hand for a few seconds and rapidly deposited in pre-warmed (40 °C) measurement cell of the spectral device.

### 2.2. Fluorescence Spectroscopy

The front face excitation synchronous fluorescence spectra were recorded by a FluoroMax-4 (Jobin–Yvon, Longjumeau, France) with a signal to noise ratio of 3000:1, equipped with a solid sample holder and a quartz cell (30 × 10 × 10 mm). During acquisition, the excitation wavelength (λex) and emission wavelength (λem) were scanned in a synchronous way with an 80 nm interval (Δλ = λex − λem) [7]. Spectra were recorded from 250 to 550 nm at 40 °C (±1 °C) every 5 min during coagulation. The temperature was controlled with a water bath equipped with a thermostat control [27]. Collections of spectral data were obtained by using the FluorEssence software (2010), version 3.5 (Horiba scientific, Jobin–Yvon, Longjumeau, France). For each formulation, three replicates were performed. Synchronous fluorescence spectra were preprocessed by Standard Normal Variate (SNV) method performed with the PLS_toolbox 8.5.2 (Eigenvector Research, Manson, WA, USA) in MATLAB 8.0.0.783 (R2012b) (The MathWorks, Natick, MA, USA). 

### 2.3. Two-Dimensional Correlation Spectroscopy 

The 2DCOS-synchronous fluorescence spectra were calculated after treatment of the collections of coagulation time-dependent dynamic spectra with 2DCOS analysis method by using a home software program in MATLAB. The 2DCOS spectra (synchronous and asynchronous) were obtained after applying to initial sample spectra an external perturbation, here coagulation time, and measuring a series of dynamic spectra and then processing these dynamic spectra by the Noda algorithm [17]. The reference spectra were chosen as the average spectrum, as previously reported by Noda and Ozaki [28]. In the present study, the 2DCOS spectral intensities are plotted as a function of wavelength variables. The two orthogonal axes of spectral variables define the 2D map or spectra, and the third axis, normal to the plane, which is the intensity. 

## 3. Results and Discussion 

### 3.1. Spectral Analysis and Comparison between Milk Formulations 

**Figure 1:**
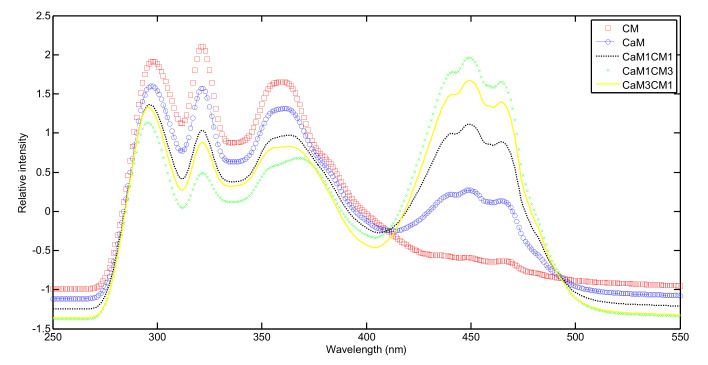
Normalized mean synchronous fluorescence spectra recorded during coagulation of CM (cow’s milk), CaM (camel’s milk) and their mixtures.

A high part of the fluorescence chemical molecules of milk samples can be revealed and identified in the synchronous fluorescence spectra. The normalized mean emission synchronous fluorescence patterns of the different milk samples recorded during coagulation are presented in Figure 1. Four bands can be observed centered on 297 nm (emission 377 nm), 321 nm (emission 401 nm), 360 nm (emission 440) and 449 nm (emission 529 nm). Those bands were previously identified, in milk and cheese [13,14], and were assigned to tryptophan, vitamin A and riboflavin compounds, respectively. The analysis of the band intensities and shapes suggested that physicochemical and molecular structure differences of the aforementioned molecules could be observed during coagulation of the different milk formulations. The analysis of the initial spectra is very useful to extract general differences between samples by observing the occurrence of fluorophores in the materials after observing, principally, the peak positions and, to a lesser extent, their shapes and intensities. However, when considering all the spectra (results not shown) recorded during coagulation of the five milk formulations (i.e., 5 formulations × 24 spectra recorded during coagulation time = a total of 120 spectra) the small differences among them make it difficult to discriminate samples only by using the conventional chemometric technique (e.g., principal component analysis) and especially when only one SFS offset (i.e., 80 nm) is used for spectral acquisition. In order to overcome this difficulty, the 2D COS method was used.

### 3.2. Analysis of 2D COS Synchronous and Asynchronous Spectra

**Figure 2:**
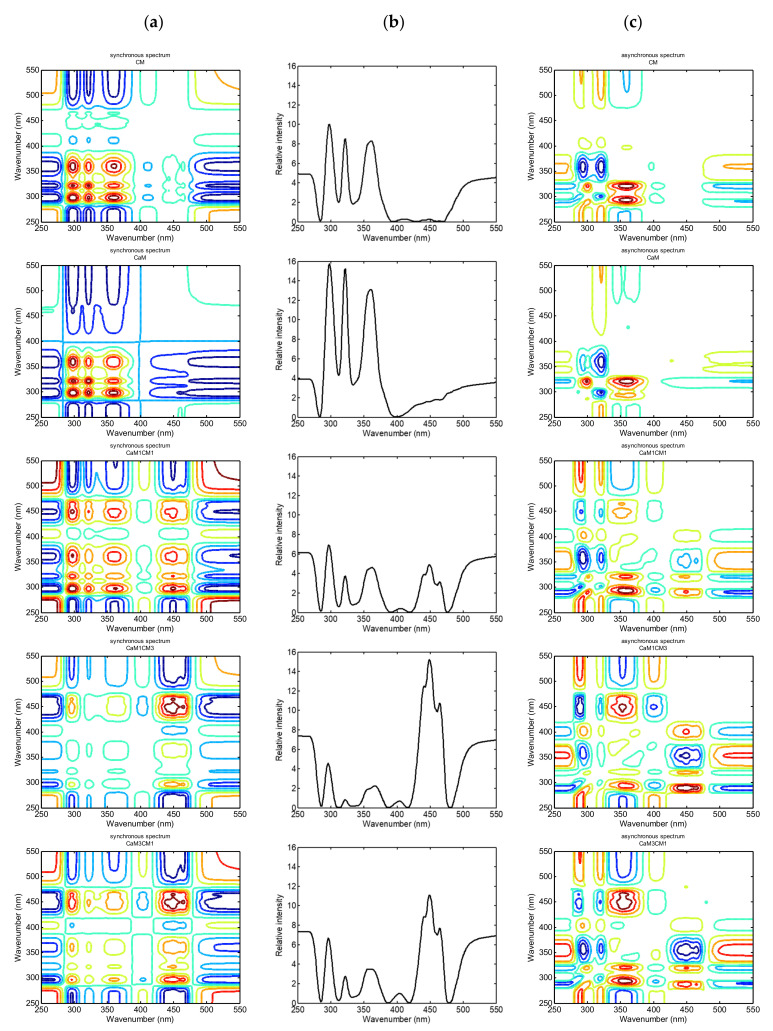
Synchronous spectra (**a**), auto-peak spectra (**b**) and asynchronous spectra (**c**).

The synchronous spectrum of 2DCOS presents in the diagonal line peaks, named auto-peaks. Those auto-peaks are the result of autocorrelation between fluorescence bands and the dynamic fluctuation. They highlight the sensitivity of fluorescence to the external perturbation, here, coagulation time. The cross-peaks located at the off diagonal revealed the relativity of intensity variations of a pair of group vibrations corresponding to their frequencies. A positive cross-peak (in red or green color area in Figure 2) indicates a simultaneous increase or decrease of different fluorescence bands under the coagulation time. The more the intensity changes between fluorescence peaks is coordinated, the stronger the cross-peak is. In contrast, a negative cross-peak (blue area color in Figure 2) represents the coordinated changes of band intensities in the opposite directions, increase versus decrease [15,17].

**Figure 3:**
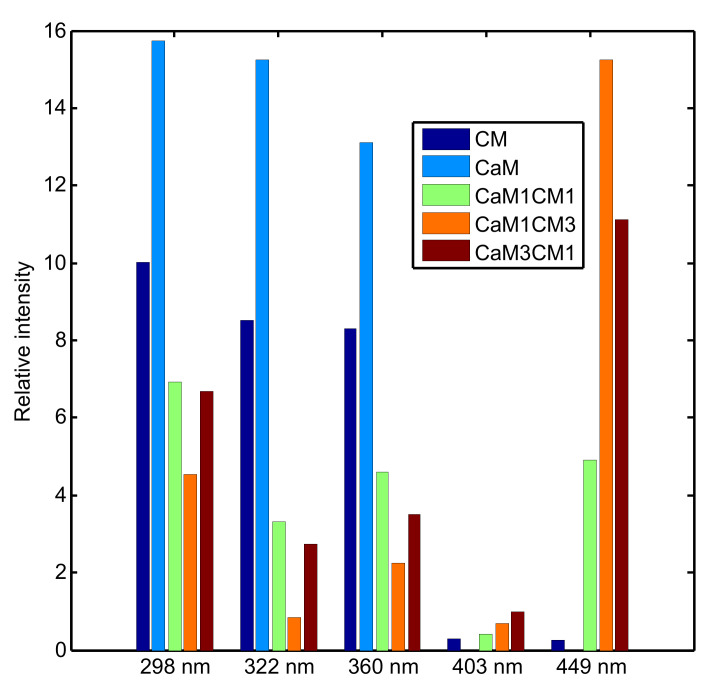
Relative auto-peak intensity of CM (cow’s milk), CaM (camel’s milk) and their mixtures.

The Figure 2a, b exhibits the synchronous 2DCOS fluorescence spectra and their respective auto-peaks spectra. The regions from 250–280 nm and 500–550 nm were excluded from the analysis because they only describe baseline modifications. Figure 3 presented the intensity of auto-peaks identified in the synchronous spectra. Figure 2a,b indicates that three major auto-peaks can be clearly identified for the CaM during coagulation, while five auto-peaks are clearly visible for the other formulations containing CM (CM, CaM1:CM1, CaM1:CM3 and CaM3:CM1). For CaM, the auto-peaks were located at 298, 322 and 361 nm. For CM, peaks identified were centered at 298, 322, 361, 412 and 449 nm. 

The peak cluster between 280 and 390 nm was present in the five formulations and the peaks were all positively auto correlated. The 449 nm peak, when present in the map, is positively auto correlated to the peak cluster located between 280 and 390 nm, suggesting synchronous modification of the peak intensities during coagulation time. Nonetheless, differences in intensities were observed between samples (Figure 3). The highest intensities were noted for CaM, then for CM, CaM1:CM1, and finally for CaM1:CM3. For the 449 nm band, the highest intensity was observed for CaM1:CM3, CaM3:CM1 and finally CM.

Concerning the band maxima of proteins, it generally reflects the average exposure of their tryptophan residues to the aqueous phase [29]. The comparison between the tryptophan position band of CaM, CM and samples containing a mixture of the two revealed a shift to lower wavelength (298 towards 296 nm). Spectral shifts have been observed because of several phenomena, such as tertiary structure change, binding of ligands and protein–protein association. In a previous study, fluorescence of tryptophan was used to monitor the structural modifications of proteins and their physicochemical environment during the coagulation process [30]. The excitation peak of tryptophan is highly sensitive to its local environment, and can be assigned to different protein conformational changes [29] during coagulation and to the gel structure differences obtained depending on milk formulation. Concerning the fluorescence of vitamin A (322 nm), it was reported that this band did not exhibit any correlation to the milk coagulation process. As reported by Boubellouta et al. [11], during the coagulation of CM, the modification of the band intensity can indicate a fluorescence transfer between tryptophan residues of caseins and vitamin A located in the membrane of fat globules. This is in agreement with an interaction of caseins with fat-globule membrane during the formation of the protein network [31,32]. 

When compared to CM and CaM, the formulations containing both CaM and CM presented peaks located at the same positions only for two bands (i.e., 322 and 449). Concerning, the band at 361 nm, this one was identified at 362, 362 and 367 for CaM3:CM1, CaM1:CM1 and CaM1:CM3 samples, respectively. This highlighted a red shift of the 361 nm band when increasing the content of CaM in the formulation. This fluorescence band could be assigned to the photochemical degradation product derived from riboflavin, lumichrome; with excitation/emission maxima generally identified at 360/450 nm in a model system [33] and 370/430 in yoghurt [34].

The small peak located at 412 nm in CM shifted to lower wavelength (403 nm) for formulations containing both CaM and CM. No literature was found that accounted with certainty for the fluorophores at the origin of this band. However, Kikugawa and Beppu [35] and Wold et al. [36] associated this peak to fluorescence from stable oxidation products, resulting from aldehydes and amino acids. The peaks centered at 403 nm presented the highest intensities for CaM3:CM1 compared to CaM1:CM3, CaM1:CM1 and finally CM samples. This peak was negatively auto correlated with the other peaks. This suggested that when this peak increased the other ones decreased during coagulation time (Figure 2b). 

Concerning the 449 nm band, it was generally assigned to riboflavin and can be used to identify protein conformation and interaction changes since it interacts with proteins [37]. 

The number, position and intensity of the auto-peaks are different among the milk formulations and can be used to differentiate molecular structure difference during their coagulation. Moreover, the profiles of the components are in agreement with the first inspection of the fluorescence landscapes, and further indicate the fact that the obtained fluorescence signal from the samples arise from tryptophan, vitamin A and riboflavin compounds. Nonetheless, in addition of identifying the synchronous relationships between fluorescence bands during coagulation, a new peak was highlighted at 403/412 nm. The results emphasized that changes in the intensity of vitamin A and riboflavin at 322 and 482 nm, respectively, can provide elements concerning molecular structure modifications taking place in the milk micelle structure [11]. The results also bring to the forefront that tryptophan and riboflavin fluorophores are relevant tracer molecules to monitor milk coagulation.

The asynchronous 2DCOS map gives the sequence modifications of the spectral band intensity at different wavelengths or λ alongside the coagulation time (external perturbation). When the perturbation is applied, a cross-peak at variable λ1 and λ2 emerges in the asynchronous 2DCOS spectra if the spectral intensity at variable λ1 changes before or after variation at variable λ2. As the intensity of the spectral bands is varying simultaneously with itself, no peak is observed along the diagonal line in the asynchronous 2DCOS map. According to Noda’s theory, the sign of the cross peaks located at λ1 and λ2 in both synchronous and asynchronous 2DCOS maps can be used to bring to the forefront the sequence of the spectral intensity variations at λ1 and λ2 [38]. If the same signs are observed in synchronous and asynchronous cross-peaks, this would highlight that the intensity change in λ1 occurs before λ2, while opposite signs in the synchronous and asynchronous cross-peaks highlight that changes in band intensity at λ2 occurs before λ1.

The asynchronous spectra further revealed the sequence of the molecular fluorescence modification during milk coagulation (structure, interaction and environment). Below the diagonal line of the asynchronous maps of the different milk formulations, five peaks were identified at 298, 322, 360, 403 and 450 nm. The different cross-peaks formed by those bands are presented in Table 1. According to Noda’s theory, the asynchronous and synchronous maps features can be analyzed as the sequence of the fluorescence modifications following the order of 360 nm (riboflavin/lumichrome) > 450 nm (riboflavin) > 403 nm (aldehydes and amino acids) > 298 nm (tryptophan) > 322 nm (vitamin A). This is consistent with the milk coagulation procedure observed after adding chymosin, hydrolysis of κ-casein, destabilization of casein micelles and aggregation [39].

## 4. Conclusions

Molecular fluorescence spectroscopy associated with 2DCOS spectroscopy was used to monitor modifications occurring during the coagulation of different milk formulations based on CM and CaM mixtures. The method demonstrated that this strategy successfully discriminates milk mixtures and monitors molecular structure modifications occurring during coagulation process.

The dissimilarities among the different formulations are precisely noticed on the synchronous 2DCOS fluorescence spectra. In addition, according to the cross-peak symbols in synchronous and asynchronous spectra, the speed of modification in the fluorescence molecules (riboflavin, protein and vitamin A) during coagulation time corroborated with common coagulation phenomena usually reported during chymosin coagulation. This research gave a promising methodology to discriminate and monitor the coagulation of milk by fluorescence and 2DCOS in an efficient, effective and non-destructive way. This method could also be proposed in the near future to identify adulteration of CaM by CM before and during processing.

## Figures and Tables

**Table 1:** The results of two-dimensional correlation spectroscopy (2DCOS) analysis of synchronous fluorescence spectra during milk coagulation: peak assignments and the signs of the cross-peaks in synchronous spectra and asynchronous spectra (shown in bracket).

	Sign of Identified Peak				
Peak Position (nm)	298 nm	321 nm	360 nm	403 nm	450 nm
298 nm	+	+	+	-	+
		(-)	(+)	(-)	(+)
322 nm		+	+	-	+
			(+)	(-)	(+)
360 nm			+	-	+
					(-)
403 nm				+	+
					(+)
450 nm					+
